# Adverse Events Related to COVID-19 Vaccines Reported in Pregnant Women in Brazil

**DOI:** 10.1055/s-0042-1755461

**Published:** 2022-09-06

**Authors:** Carla Dinamerica Kobayashi, Victor Bertollo Gomes Porto, Martha Elizabeth Brasil da Nóbrega, Cibelle Mendes Cabral, Tiago Dahrug Barros, Cecília Maria Roteli Martins

**Affiliations:** 1 General Coordination of the National Immunization Program, Ministério da Saúde, Brasília, DF, Brazil; 2 Centro Universitário Faculdade de Medicina do ABC, Santo André, SP, Brazil

**Keywords:** COVID-19 vaccines, adverse event, vaccine-preventable diseases, pregnant women, Brazil, vacinas contra COVID-19, evento adverso, doenças preveníveis por vacina, gestantes, Brasil

## Abstract

Regulations for the vaccination of pregnant women in Brazil occurred in March 2021. Despite the absence of robust data in the literature on the coronavirus disease 2019 (COVID-19) vaccinations in pregnant women, it is understood that the benefit-risk ratio tends to be favorable when considering the pandemic and the high burden of the disease. However, it is still important to monitor for Events Supposedly Attributable to Vaccination or Immunization (ESAVI) and to draw safety profiles of the different platforms used in pregnant and postpartum women. The present study aims to describe the main characteristics of ESAVIs related to COVID-19 vaccines occurring in pregnant women in the first months of the vaccination campaign in Brazil. During the evaluation period, 1,674 notifications of ESAVIs in pregnant women were recorded, and 582 notifications were included for the analysis. Of the 582 ESAVIs identified, 481 (82%) were classified as non-serious adverse events and 101 (17%) as serious adverse events. Ten deaths were identified, including one death which was considered to be causally related to the vaccine. The other nine maternal deaths had causality C, that is, without causal relationship with the vaccine, and most were due to complications inherent to pregnancy, such as pregnancy-specific hypertensive disorder (PSHD) in 4 cases and 3 due to COVID-19. Despite some limitations in our study, we believe it brings new insights into COVID-19 vaccines in this group and will add to the available evidence.

## Resumo

As determinações vacinação nas gestantes foram estabelecidas em março de 2021, no Brasil. Apesar da ausência de dados robustos na literatura da vacinação contra *coronavirus disease 2019* (COVID-19) nesse grupo, entende-se que a relação de benefício versus risco tende a ser favorável considerando a situação pandêmica e a elevada carga de doença, tendo justificado o uso dessas vacinas em ampla escala nas gestantes de todo o mundo. Entretanto, o monitoramento dos eventos adversos pós vacinação (EAPVs) torna-se ainda mais importante para traçar um perfil de segurança das diferentes plataformas nas gestantes e puérperas. O presente estudo tem como objetivo descrever as principais características dos EAPVs contra COVID-19 ocorridos nas gestantes nos primeiros meses de campanha da vacinação no Brasil. Foram identificadas 1.674 notificações em gestantes, com a inclusão de 582 EAPVs analisados. Dos 582 EAPVs identificados, 481 (82%) foram classificados como eventos adversos não-graves e 101 (17%) como eventos adversos graves, sendo 10 (9,9%) destes referentes aos óbitos. Apenas um caso de óbito materno teve relação causal com a vacinação comprovada (causalidade A1), e foi secundário à síndrome trombocitopênica trombótica (TTS) após a vacina AstraZeneca/Fiocruz. Os outros nove óbitos maternos tiveram causalidade C, ou seja, sem relação causal com a vacina, e a maioria por complicações inerentes à gravidez, como a doença hipertensiva específica da gestação (DHEG) e COVID-19. Apesar de algumas limitações em nosso estudo, acreditamos que ele traz dados importantes sobre as vacinas COVID-19 neste grupo aumentando as evidências disponíveis.

## Introduction

Since the beginning of the coronavirus disease 2019 (COVID-19) pandemic in December 2019, in China, up until February 2022, the world has seen multiple epidemic waves leading to over 5.6 million deaths and 376 million confirmed cases worldwide. 1 Brazil has been particularly hit by the pandemic, accounting for over 628 thousand deaths and 25.6 million cases until February 2022. 2

Pregnant women and those in the postpartum period up to 45 days after delivery are associated with higher risk for the development of severe disease caused by COVID-19, including a higher risk of admission to the intensive care unit, need for mechanical ventilation, and even death when compared to non-pregnant women of the same age. Additionally, there is an increased risk for unfavorable obstetric outcomes resulting from the disease, such as premature delivery and fetal death. 3 4 The mortality rates from COVID-19 was higher in pregnant women in Brazil than in other countries, and by February 2022, 1,978 maternal deaths due to COVID-19 had been confirmed in the country. 5

The health, social, and economic crisis unleashed by the COVID-19 pandemic led to massive investments in vaccine research, leading to the successful development of a number of vaccines against the virus in record time. In December 2020, approximately 1 year after the virus was first identified, the World Health Organization (WHO) approved the emergency use of the first COVID-19 vaccine. Presently, 10 different COVID-19 vaccines have been approved for clinical use by the World Health Organization (WHO), including vaccines with new platforms, such as non-replicating viral vector vaccines (AstraZeneca and Janssen) and messenger RNA (mRNA) vaccines (Pfizer/Wyeth and Moderna) and consecrated platforms such as the aluminum-hydroxide-adjuvanted, inactivated whole virus vaccine produced by Sinovac, the CoronaVac vaccine.

In Brazil, vaccination against COVID-19 started in January 2021. Considering the lack of data regarding the safety and efficacy/effectiveness of COVID-19 vaccination in pregnant and postpartum women, these groups were not included during the first phases of the Brazilian vaccination strategy and were only included as a priority group after extensive discussions about the risks and benefits. Regulations for the vaccination of pregnant women in Brazil were published in the technical note of March 15, 2021, from the Brazilian Ministry of Health (MoH). 6

Despite the absence of robust data in the literature on the COVID-19 vaccinations in pregnant women, it is understood that the benefit-risk ratio tends to be favorable when considering the pandemic and the high burden of disease. Therefore, large-scale use of these vaccines in pregnant women worldwide is justified. However, it is still important to monitor for Events Supposedly Attributable to Vaccination or Immunization (ESAVIs) and to draw safety profiles of the different platforms used in pregnant and postpartum women.

The reporting of serious adverse events (SAEs) is mandatory in Brazil and any healthcare professional which identifies a SAE is required to report the event to the National Immunization Program (NIP) within 24 hours. Brazil has a well-established surveillance system for ESAVIs, a partnership between the National Agency for Sanitary Surveillance (Anvisa) and the NIP. After being notified, the adverse events are investigated by local surveillance teams and evaluated for causality assessment.

Prior to the beginning of the COVID-19 vaccination strategy, the NIP adopted a new online information system for the reporting of ESAVIs, e-SUS Notifica, which allows for the notification of an ESAVI by any healthcare professional directly. Locations with limited internet access are required to fill an ESAVI reporting form, which is sent to a centralized center in order to be typed into the online system.

The present study aims at describing the main characteristics of ESAVIs related to COVID-19 vaccines occurring in pregnant women and reported to the Brazilian NIP in the first months of the vaccination campaign in Brazil.

## Methods

This is a descriptive observational study of a case series of ESAVIs in pregnant women reported between January 18 and August 1, 2021, in Brazil.

All cases of ESAVIs in pregnant women reported to the Brazilian MoH information system for ESAVIs, e-SUS Notifica, between January 18 to August 1, 2021, in Brazil.

The definitions used are those recommended by the World Health Organization (WHO) 7 and described in the Brazilian Guidance for Epidemiological Surveillance of Adverse Events Following Vaccination 8 :

1) Events Supposedly Attributable to Vaccination or Immunization (ESAVI): any unwanted medical occurrences after vaccination, not necessarily causally related to the use of a vaccine or other immunizing agents (immunoglobulins and heterologous serum). An ESAVI can be any unwanted or unintended event, that is, symptom, disease, or abnormal laboratory finding (WHO, 2016). 7
2) Serious adverse event (SAE): any clinically relevant event that (i) requires hospitalization, (ii) may compromise the patient, that is, cause risk of death and require immediate clinical intervention to prevent death, (iii) causes significant dysfunction and/or permanent incapacity, (iv) results in congenital anomaly, and (v) causes death.
3) Non-serious adverse event (NSAE): any other event that does not meet the SAE criteria.
4) Immunization error (programmatic): any avoidable event that can cause or lead to inappropriate use of immunizing agents and/or harm to the patient.
5) Causality classification: A1 (vaccine product-related reaction [as per published literature]), A2 (vaccine quality defect-related reaction ), A3 (immunization error-related reaction), A4 (immunization anxiety-related reaction [ISRR]), B1 (temporal relationship is consistent but there is insufficient definitive evidence for vaccine causing event [may be new vaccine-linked event]), B2 (qualifying factors result in conflicting trends of consistency and inconsistency with causal association to immunization), C (coincidental underlying or emerging condition(s), or condition(s) caused by exposure to something other than vaccine ), and D (unclassifiable).

For the present study, data related to ESAVIs such as: individual characteristics (age, self-reported race, gestational age/trimester, comorbidities), vaccination data, and ESAVI description data (dose type, vaccine type, place of vaccination, ESAVI classification [severe, not severe], type of ESAVI) were extracted from the e-SUS Notifica information system. All SAEs included were revised by medical doctors from the NIP.

Vaccination data (dose by vaccine type) was extracted from the National Network of Health-Related Data (RNDS, in Portuguese) and National Immunization Program Information System (SIPNI, in Portuguese).

To identify vaccinated pregnant women, the information regarding the priority group for vaccination and the variables of pregnancy status identified in the SIPNI and RNDS systems were considered. Data extracted from both systems were compiled in a unified dataset and deduplicated. However, it should be noted that the variable for pregnancy status was unavailable in the information systems at the beginning of the vaccination campaign and was added as a variable during the campaign. Therefore, this data does not represent the totality of vaccinated pregnant women and cannot be used to calculate the direct incidence of ESAVI occurrence in this group.

Thus, the calculation of ESAVI incidence in the group of pregnant women was performed after cross-referencing the individual taxpayer registry number (CPF) from the combined vaccination database of vaccinated pregnant women (RNDS and SIPNI) and the ESAVI notification data from “e-SUS Notifica”. This method allowed us to include only ESAVIS in pregnant women with known pregnancy status on the vaccination information system as the numerator, minimizing selection biases in the estimates of incidence. The incidence of events was calculated per 100,000 doses administered. Additionally, data regarding ESAVIs from the state of São Paulo in Brazil are not entered in “e-SUS Notifica”, hence data regarding this state was not included in the analysis.

Data analysis was performed using descriptive statistics, with relative and absolute frequency measurements. For data processing, the Pandas Library for Python version 1.1.3 was used, as well as the programs EpiInfo TM 7.2.3.1 and Microsoft Excel 2016 (Microsoft Corp., Redmond, WA, USA).

## Ethical Aspects

This study was approved by the National Research Ethics Committee (CONEP) CAAE number 53592021.8.0000.0008. The criteria of confidentiality and non-disclosure of information that could identify the individuals included in the databases were respected.

## Results

During the evaluation period, 678,025 doses of COVID-19 vaccines administered to pregnant women were registered (excluding the state of São Paulo), distributed as described in Table 1 , with the Pfizer/Wyeth vaccine being the most widely administered vaccine (395,253 doses).

**Table 1:** Distribution of doses of vaccines against coronavirus disease 2019, applied in pregnant women

Vaccine	First dose	Second dose	Total
AstraZeneca	56.362	5.966	62.328
Pfizer	386.077	9.176	395.253
Sinovac	140.651	77.795	218.446
Janssen	1.998	−	1.998
Total	585.088	92.937	678.025

**Fig. 1:**
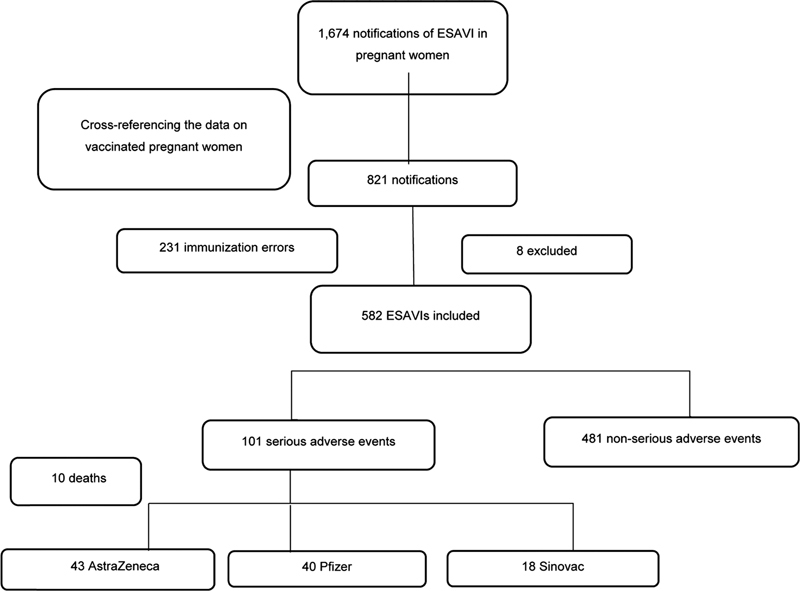
Flowchart of Events Supposedly Attributable to Vaccination or Immunization (ESAVIs) related to coronavirus disease 2019 (COVID-19) vaccines in pregnant women.

In the same period, 1,674 notifications of ESAVIs in pregnant women were recorded in e-SUS Notifica. After cross-referencing the data on vaccinated pregnant women and ESAVIs data, 821 notifications were included for the analysis (231 immunization errors and 582 adverse events), as represented in Figure 1 .

Of the 582 ESAVI-s identified, 481 (82%) were classified as NSAE, and 101 (18%) as SAEs, 10 (9.9%) of which resulted in deaths ( Figure 1 ). The incidence of SAEs was 14.9 per 100,000 doses administered. The vaccine with the highest incidence of SAEs was the one by AstraZeneca, with 68.9 events per 100,000 doses, as described in Table 2 .

**Table 2:** Events Supposedly Attributable to Vaccination or Immunization in pregnant women according to severity and vaccine type, reported to e-SUS Notifica

Adverse event	AstraZeneca		Pfizer		Sinovac		Janssen		Total	
	n	*Incid.	n	*Incid.	n	*Incid.	n	*Incid.	n	*Incid.
Serious	43	68.9	40	10.1	18	8.2	0	0.0	101	14.9
Death	3	4.8	4	1.0	3	1.4	0	0.0	10	1.5
Non-serious	342	548.7	103	26.1	35	16.0	1	50.1	481	70.9
Total	385	617.7	143	36.2	53	24.3	1	50.1	582	85.8

Abbreviation: Incid., number of reported events per 100 thousand doses administered.

The age distribution was similar among pregnant women in the three vaccination groups, with the majority being between 20 and 34 years of age (438/582). The mean age was also similar between the SAEs and NSAEs, with a mean of 29.5 and 27.7 years, respectively. Most of the pregnant women's reported races were white (268/582), as self-declared when using the notification system. The Southeast (212/582) and South (195/582) regions accounted for most of the notifications ( Table 3 ). Systemic arterial hypertension (SAH) and diabetes mellitus (DM) were the most prevalent morbidities among pregnant women. Most ESAVis were reported during the 3 ^rd^ trimester of pregnancy (51.4%), followed by the 2 ^nd^ trimester (29.4%). Only 12 (2.1%) ESAVIs occurred after the 2 ^nd^ dose ( Table 3 ).

**Table 3:** Overall characteristics related to Events Supposedly Attributable to Vaccination or Immunization

	AstraZeneca (n = 385)		Pfizer (n = 143)		Sinovac (n = 53)		Janssen (n = 1)		Total (n = 582)	
	n	%	n	%	n	%	n	%	n	%
Severity										
Serious	43	11.2	40	28	18	34	0	0	101	17.4
Non serious	342	88.8	103	72.0	35	66	1	100	481	82.6
Age (years)										
15–19	25	6.5	16	11	0	0	0	0	41	7.0
20–24	93	24.2	38	27	22	42	1	100	154	26.5
25–29	118	30.6	26	18	9	17	0	0	153	26.3
30–34	92	23.9	30	21	9	17	0	0	131	22.5
35–39	47	12.2	26	18	9	17	0	0	82	14.1
40–44	10	2.6	6	4.2	4	7.5	0	0	20	3.4
45–49	0	0.0	1	0.7	0	0			1	0.2
Self-reported skin color										
White	184	47.9	61	43	23	44	0	0	268	46.0
Brown	141	36.6	46	32	25	47	1	100	213	36.6
Black	16	4.1	17	17	4	7.5	0	0	37	6.4
Yellow	1	0.2	1	0.7	0	0	0	0	2	0.3
Ignored	43	11.2	18	13	1	1.9	0	0	62	10.7
Regions										
North	13	3.4	4	2.8	5	9.4	0	0	22	3.8
Northeast	67	17.4	23	16	3	5.6	0	0	93	16.0
Middle West	41	10.6	8	5.6	6	11	1	100	56	9.6
Southeast	130	33.7	68	47.5	18	34	0	0	216	37.1
South	134	34.8	40	28	21	40	0	0	195	33.5
Gestational age (trimester)										
1° (0–12 weeks)	74	19.2	35	25	13	25	0	0	122	21.0
2° (13–23 weeks)	113	29.4	29	20	10	19	1	100	153	26.3
3° (24–40 weeks)	198	51.4	79	55	30	57	0	0	307	52.7
Previous conditions										
Systemic arterial hypertension (SAH)	18	4.6	7	4.8	4	7.5	0	0	29	4.9
Diabetes mellitus (DM)	18	4.6	8	5.5	0	0	0	0	26	4.4
Obesity	2	0.0	4	2.8	0	0	0	0	6	1.5
More than one (SAH/DM/Obesity)	4	1.0	4	2.8	0	0	0	0	8	0.9
Asthma	6	1.5	4	2.8	1	2.3	0	0	11	1.3
Chronic kidney disease	1	0.2	0	0.2	0	0	0	0	1	0.2
Vaccine dose										
1 ^st^ dose	384	99.7	139	97.2	46	86.8	1	100	570	97.9
2 ^nd^ dose	1	0.0	4	2.8	7	13	0	0	12	2.1

Unspecific systemic symptoms such as fever, myalgia, and headache were the most commonly reported NSAEs (87.5%), mostly associated with the AstraZeneca vaccine (95%). Seven cases of genital bleeding were reported among the NSAEs, all of which were considered to be temporarily related to the vaccine but without sufficient evidence in the literature to establish a causal relationship. Coronavirus disease 2019 infection was the most frequently reported coincidental event ( Chart 1 ).

**Chart 1:** Non severe Events Supposedly Attributable to Vaccination or Immunization against coronavirus disease 2019 in pregnant women according to causality classification, event, and vaccine, reported to e-SUS Notifica

Causality assessment	n	Adverse event	Vaccine
A.1- Vaccine product-related reaction (As per published literature)	6	Local reactions -Pain/erythema/edema	3 AstraZeneca
			2 Pfizer
	11	Exanthema/Rash/Pruritus	1 AstraZeneca
			5 Pfizer
			5 Sinovac
	421	Unspecific systemic symptoms (fever, myalgia, headache)	395 AstraZeneca
			76 Pfizer
			19 Sinovac
			01 Janssen
	3	Tachycardia/Hypertensive crisis	2 Pfizer
			1 Sinovac
	4	Abdominal pain	3 AstraZeneca
			1 Sinovac
B.1 - Temporal relationship is consistent but there is insufficient definitive evidence for vaccine causing event	7	Genital bleeding	2 AstraZeneca
			2 Pfizer
			3 Sinovac
C - Coincidental underlying or emerging condition(s), or condition(s) caused by exposure to something other than vaccine	21	Covid-19	5 AstraZeneca
			11 Pfizer
			2 Sinovac
	4	Urinary tract infections	2 Pfizer
			2 AstraZeneca
	1	Asthma	1 Pfizer
	1	Deep venous thrombosis	1 AstraZeneca
	2	Edema	1 Pfizer
			1 Sinovac

During the evaluation period, 101 SAEs were reported, 43 (42.6%) after the AstraZeneca vaccine, 40 (39.6%) after the Pfizer vaccine, and 18 (17.8%) after the Sinovac vaccine. Infection by COVID-19 was the most frequent non-pregnancy-related adverse event, with 14 cases and 3 maternal deaths. Only one case of maternal death had a proven causal relationship with vaccination (causality A1) and was secondary to thrombotic thrombocytopenic syndrome (TTS) after the AstraZeneca/Fiocruz vaccine. The other nine maternal deaths had causality C, that is, without causal relationship with the vaccine, and most were due to complications inherent to pregnancy, such as pregnancy-specific hypertensive disorder (PSHD) in 4 cases and 3 due to COVID-19 ( Table 4 ). Pregnancy-related adverse events corresponded to most of the SAEs; the most frequent being fetal death (26/101), with 3 of these cases classified as B1 and occurred in the 3 ^rd^ (14/26) and 2 ^nd^ trimesters (12/26). First trimester abortion was the 2 ^nd^ most prevalent (2/101) SAE, all with causality C, as shown in Table 4 .

**Table 4:** Severe Events Supposedly Attributable to Vaccination or Immunization against coronavirus disease 2019 in pregnant women according to causality classification, diagnosis, and vaccine

	AstraZeneca (n = 43)	Pfizer (n = 40)	Sinovac (n = 18)	Total (n = 101)
First trimester abortion				
Causality C	9	12	3	24
Fetal death				
Causality B1	1	2	0	26
Causality C	8	11	2	3
Causality D	0	1	1	220
Maternal death				
Causality A1 (TTS*)	1	0	0	1
Causality C (4 PSHD*, 1 stroke, 1 coagulation disorders)	2	2	2	6
COVID-19	0	2	1	3
COVID-19				
Causality C	5	5	4	14
Deep venous thrombosis (DVT)				
Causality C	1	1	0	2
Stroke				
Causality B1	0	1	1	2
Other neurologic conditions (seizure/facial palsy)				
Causality B1	0	2	1	3
Immediate hypersensitivity reactions				
Causality A1	0	2	1	3
Unspecific systemic symptoms				
Causality A1	4	0	0	4
Genital bleeding				
Causality B1	3	0	1	4
Preterm labor				6
Causality B1	2	0	0	2
Causality C	3	0	1	4
Amniotic fluid loss/oligohydramnios				
Causality C	1	0	1	2
Pregnancy-specific hypertensive disorder (PSHD)				
Causality C	1	0	0	1
Other diagnosis				
Causality C	2	1	0	3

Abbreviations: A1, vaccine product-related reaction (as per published literature); B1, temporal relationship is consistent but there is insufficient definitive evidence for vaccine causing event (may be new vaccine-linked event); C, coincidental underlying or emerging condition(s), or condition(s) caused by exposure to something other than vaccine; D, unclassifiable; TTS, thrombocytopenic thrombotic syndrome; PSHD, pregnancy-specific hypertensive disorder.

## Discussion

The present study described the occurrence of ESAVIs in pregnant women vaccinated against COVID-19 which were reported to e-SUS Notifica during the first 6 months of the vaccination campaign in Brazil, including over 678 thousand vaccinated pregnant women and 582 events. Most events were non serious (82.6%), although 10 deaths were reported, and only 1 death was considered to be causally related to the vaccine.

The Southeast and South regions were responsible for ⅔ (70%) of the notifications (excluding the state of São Paulo). The exclusion of the state of São Paulo was required due to the adoption of a different notification system in that state. Such exclusion led to the loss of an important dataset considering that it is the most populous state in the country, with 46 million inhabitants as of 2020, and administered more than 35 million doses during the same period of the study.

In addition to the exclusion of the state of São Paulo, only the notifications of women with confirmed pregnancy status in the vaccinated database were analyzed for the consistent calculation of event incidences. This selection bias likely led to an underrepresentation of pregnant women vaccinated during the first trimester, since it is likely that these women were not aware of their pregnancy status when receiving the vaccine.

The majority of our data correspond to the 1 ^st^ vaccine dose since only 13.7% of the vaccinated individuals had received their 2 ^nd^ dose during the analysis period. Likewise, only 12 events were reported after the 2 ^nd^ dose, most of which were associated with the Sinovac vaccine (n = 7, 58.3%). This is likely due to the different recommended intervals for each vaccine as indicated by the NIP, which was initially 12 weeks for the AstraZeneca and Pfizer vaccines compared with 28 days for the Sinovacas well as to the fact that pregnant women were only considered a priority group from March 20, 2021. 9

The AstraZeneca COVID-19 vaccine showed higher incidence of ESAVIs, 617.6 events per 100,000 doses administered in pregnant women, mostly non-severe unspecific systemic symptoms. This higher incidence for the AstraZeneca vaccine was also observed in the general population, 134.9 events per 100 thousand administered doses versus 92 events for the Sinovac vaccine, as described in the special epidemiological bulletin 5 from the same period.

Most pregnant women were aged between 20 and 34 years (75% in the AstraZeneca and Sinovac groups and 65% in the Pfizer group). The 3 ^rd^ trimester was the most frequent trimester at the time of vaccine administration for all vaccines. More than ⅔ of the described events (421/582) were due to unspecific systemic symptoms, such as headache, myalgia, fever, and nausea, in all vaccines. These are expected adverse events which had been previously described in clinical trials by the manufacturers and are likely related to the vaccine itself. These data are similar to recently published studies with mRNA 10 vaccines.

Coronavirus disease 2019 infection was the second most frequent NSAE, which reflects the high risk of infection during the study period, making it likely that a proportion of the NSAE which were reported due to undiagnosed COVID-19 cases. For the present study a diagnosis of COVID-19 was made with a positive molecular or antigen tests and those with descriptions of specific symptoms such as anosmia and/or dysgeusia, according to the case definitions for COVID-19 adopted in the Brazilian Epidemiological Surveillance Guide. 11 Limitations regarding testing availability in the 1 ^st^ months of the pandemic likely led to an underestimation of the true number of COVID-19 cases in our study group.

Among the 101 SAE reported there were 10 cases of maternal deaths. Only one death was classified as a vaccine product-related reaction according to WHO causality assessment algorithm, resulting from a confirmed case of thrombocytopenic thrombotic syndrome (TTS) related to the AstraZeneca vaccine. This led to an estimated incidence of 1.6 cases of TTS per 100,000 doses of AstraZeneca vaccine administered to pregnant women. Such incidence is consistent with the incidence described in the general population of roughly one case every 100,000 doses. 5 Thrombocytopenic thrombotic syndrome is an extremely rare syndrome which has been described as likely caused by non-replicating adenoviral vaccines such as AstraZeneca and Janssen. Despite its severity, it is generally understood that the risk-benefit of vaccination is still highly favorable.

The incidence of SAEs post AstraZeneca vaccine was low, 68.9 per 100 thousand doses. Although we do not use viral vector vaccine in our country, after these results it seems to be plausible the vaccination with AstraZeneca in countries that do not have other platforms available.

The other SAEs leading to maternal deaths were classified as “C,” being coincident or inconsistent (i.e., they had causes other than the vaccine as triggering factors), including 3 cases of COVID-19 initiating on the first few weeks immediately after vaccination. These results strengthen our recommendation to vaccinate pregnant women, especially due to the greater risk of complications inherent to the disease. A total of 14 cases of severe COVID-19 were reported, amounting for 13.8% of the 101 SAEs. Coronavirus disease 2019 infection was indeed responsible for the majority of the non-obstetric events.

Among pregnancy-specific events, fetal death and first trimester abortion (up to the 13th week of pregnancy) were the most frequent SAEs, with an incidence of 3.8 and 3.5 cases per 100,000 doses, respectively. Spontaneous abortion, fetal death, and premature birth are obstetric complications inherent to pregnancy. Systematic reviews point to an expected gestational loss of around 15% for the total of confirmed 12 pregnancies. According to data extracted from the Brazilian National Information System on Live Births (SINASC), Brazil had a 3.4% spontaneous abortion rate before the COVID-19 pandemic and an 11% preterm birth rate per year. 13 Spontaneous abortion was also among the most frequent adverse events reported with other vaccines such as influenza. 14 Our study period did not account for the entirety of the pregnancy duration, hence it is not possible to draw direct comparisons; nevertheless, such low reporting rates are likely within the expected.

Vaccination against COVID-19 was initiated in pregnant women, even in the absence of clinical trials or robust safety data in literature. This recommendation was justified mainly by the higher risk of hospitalizations, deaths, and obstetric complications associated with the COVID-19 infection in pregnant women, that is, a favorable risk-benefit ratio for vaccination. Additionally, the existence of preliminary phase 4 studies with the Pfizer vaccine did not warn of adverse outcomes in vaccinated 10 pregnant women.

The occurrence of one case of TTS related to the AstraZeneca vaccine led to the interruption of the use of adenoviral-vectored vaccines in pregnant women in Brazil. Although, we understand that the occurrence of such case does not suggest an increased risk of occurrence in pregnant women, considering that the incidence was within expected. Also, in countries with low availability of alternative vaccine platforms for pregnant women, it is likely that the use of adenoviral-vectored vaccines in this population can be justified.

## Conclusion

Events resulting in unfavorable obstetric outcomes for pregnant women or fetuses are considered severe events of special interest, since data in literature are limited in this population. Surveillance of these events, even if passive, is essential to profile the safety of vaccines in pregnant women. Despite some limitations in our study, we believe it brings new insights into COVID-19 vaccines in this group and will add to the available evidence.
